# Multiple Large Tumefactive MS Plaques in a Young Man: A Diagnostic Enigma and Therapeutic Challenge

**DOI:** 10.1155/2012/363705

**Published:** 2012-07-12

**Authors:** Hossein Kalanie, Ali Amini Harandi, Reza Bakhshandehpour, Daryoosh Heidari

**Affiliations:** ^1^Mehr Hospital, Shahid Beheshti University of Medical Sciences, Tehran, Iran; ^2^Loghman Hakim Hospital, Shahid Beheshti University of Medical Sciences, Tehran, Iran; ^3^Shahid Beheshti University of Medical Sciences, Tehran, Iran

## Abstract

Tumefactive demyelinating lesion is defined as large solitary demyelinating lesion with imaging characteristics mimicking neoplasm. These atypical features include size more than 2 cm, mass effect, edema, and/or ring enhancement. Distinguishing tumefactive lesions from other etiologies of intracranial space occupying lesions is essential to avoid inadvertent surgical or toxic chemotherapeutic intervention. Symptoms are generally atypical for multiple sclerosis (MS) and usually related to the pressure of a focal mass lesion without a history of MS. The clinical presentation and MRI appearance of these lesions often lead to biopsy. Here, we present a young man with fulminating neurological symptoms and multiple large tumefactive lesions on either hemisphere. Since patient and parents were not agreed on brain biopsy, a course of steroid therapy was commenced which ended to considerable improvement and confirmed the diagnosis of tumefactive MS. Thirteen months later, he experienced another relapse when his treatment was continued by weekly intramuscular injection of interferon b1a (Avonex). Two further MRIs showed shrinkage of tumefactive plaques and resolution of edema in the periphery of lesions.

## 1. Introduction

Multiple sclerosis (MS) is a chronic inflammatory demyelinating disease characterized by heterogeneity in clinical symptoms and evolution which sometimes may present as large demyelinating lesions, which may simulate intracranial neoplasms [1, 2]. These are called tumefactive multiple sclerosis (TMS) and may cause a diagnostic enigma for both clinicians and radiologists. The occurrence of tumor-like demyelination is reported rare and more commonly occurs in women and young adults [3].

Distinguishing TMS from, infection, abscess and malignancy especially glioma is critical for proper patient management and to avoid unnecessary medical or surgical interventions which sometimes results in unnecessary and harmful surgical resection, radiation therapy, or drainage [4–8], while in most cases it carries a benign prognosis as a precursor of multiple sclerosis. Acute manifestations may improve often by treatment with high-dose intravenous methylprednisolone or other immunosuppressive agents. Decompressive hemicraniectomy has been shown to be effective at controlling rare cases with high intracranial pressures associated with severe cerebral swelling [9].

In this paper, we present a case of TMS in an adult young man with multiple tumefactive lesions and relapse.

## 2. Case Report

At April 2008, an 18-year-old man was referred by a neurologist to our department with 6-day history of progressive deteriorating neurologic symptoms and an abnormal brain magnetic resonance imaging (MRI). His problem has begun with weakness and paresthesia in the right upper extremity with gradual extension to left arm and both lower limbs. On the third day, he felt haziness of vision in his right eye and pain on lateral gaze. In the same day, he also experienced few attacks of grand mal seizure. On neurological evaluation upon admission, he was well-oriented gentleman looking apprehensive and concerning about his symptoms. He was afebrile with normal general physical examination. Positive findings in neurological evaluation included visual acuity of 20/200 in right eye, with central scotoma and signs of papillitis on fundoscopy, jerk nystagmus on lateral gaze, decreased power in extremities (3/5 in right and 4/5 in left), +1 deep tendon reflexes, bilateral Babinski sign, and mild ataxic gait with normal superficial and deep sensations.

While patient was started on Valproate- (Depakine Chrono 500 mg twice per day) obtained lab data revealed below results: CBC, blood sugar (BS), serum electrolytes, liver and kidney function tests, and urine analysis all were normal and ESR was 15 mm. Serology for HIV, HCV, EBV, HZV, borrelia, and toxoplasma was negative so was a tuberculosis skin test and collagen vasculitis work up. Chest X-ray was normal. Cerebrospinal fluid (CSF) analysis showed 2 RBC, 0 WBC, protein of 125 mg/dL, sugar 50 mg/dL (BS: 110), LDH: 43 U/mL, and positive oligoclonal band (OCB). Visual-evoked potentials (VEP) test was prolonged in right eye so was the left-sided sensory-evoked potentials (SEPs).

**Figure 1:**
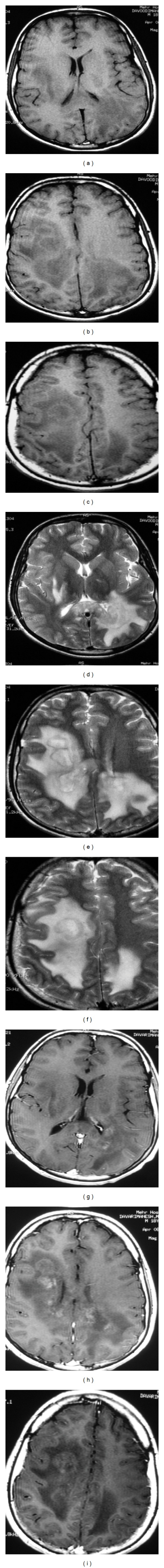
April 2008, axial T1WI ((a), (b), and (c)) and T2WI ((d), (e), and (f)) demonstrate multiple round-shaped lesions with low T2 and iso T1 margin and central low T1 and high T2 signal intensity in right centrum semiovale region and perileft occipital horn white matter, extending to splenium of the corpus callosum and are associated with peripheral vasogenic edema but mo mass effect or midline structure shift was seen. On postcontrast T1 W images ((g), (h), and (i)), heterogeneous enhancement was noted in lesions.

Brain MRI revealed multiple large round-shaped lesions with central low T1, high T2 signal high-intensity signals and marginal low T2, iso T1 signals in right centrum semiovale, and perileft occipital horn white matter extending to splenium of corpus callosum, which were associated with sever peripheral vasogenic edema but no mass effect and no midline structural shift (Figures 1(a)–1(f)). In postcontrast T1W images, heterogeneous enhancement was noted in lesions (Figures 1(g)–1(i)). Result of cervical MRI was normal.

These findings brought several differential diagnoses such as acute disseminated encephalomyelitis, multifocal glioma, metastasis, vasculitis, lymphoma, lymphoproliferative diseases, and TMS under consideration. At this stage for proper management of the patient, a biopsy was suggested which was refused by the patient and his parents, so we decided to treat the patient empirically with the diagnosis of TMS in spite of clinical (seizure) and MRI red flag signs [10]. This decision was made on the basis of the presence of papillitis, normal blood tests, prolonged VEP and SEP, presence of OCB, and no shift in midline structure in spite of large lesions.

He received a 7-day course of 1 gr daily intravenous methylprednisolone (IVMP). This was followed by further 2 weeks of 75 mg daily oral prednisolone (OPN) with tapering and discontinuation of the drug in the third week. From the third day of IVMP, he begun improving and discharged by the end of OPN therapy with mild right-sided weakness.

Thirteen months later he was returned with left optic neuritis, received 5-day course of IVMP, and with respect to 2nd MRI done at August 2008, he was discharged on weekly intramuscular injection of Interferon beta1a (Avonex). So far, he has been stable and no further relapse has been reported.

**Figure 2:**
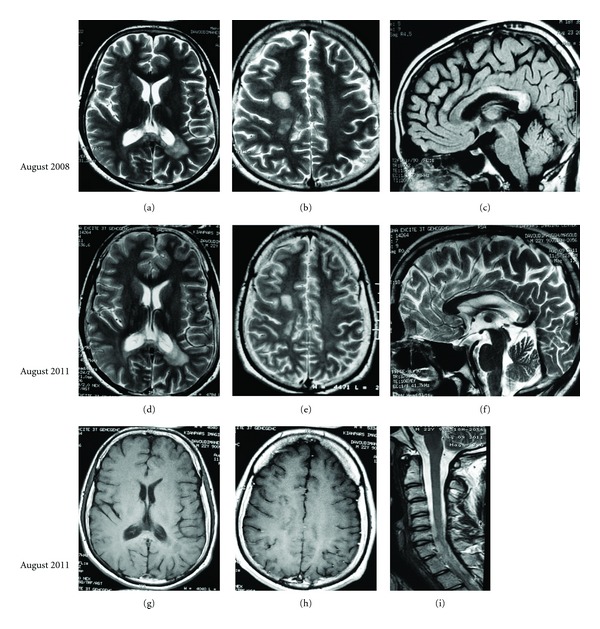
August 2008 (upper row): axial T2WI ((a) and (b)) and sagittal FLAIR (c) and August 2011 (middle row) axial T2WI ((d), (e), and (f)) demonstrate sequelae of lesions as multifocal high T2 signal intensities in right centrum semiovale area and peri-left-occipital horn white matter, involving also splenium and posterior body of the corpus callosum which were stable and the peripheral vasogenic edema is gone. August 2011 (lower row) postcontrast axial T1 W images ((g) and (h)) and sagittal cervical spine T2 WI (i), no enhancement in lesion or abnormal signal intensity in cervical cord were seen.

 Two further MRI studies were performed at August 2008 (Figures 2(a)–2(c)) and August 2011 (Figures 2(d)–2(f)). They demonstrated sequelae of previous lesions as high T2 signal intensities with resolution of their peripheral vasogenic edema. The lower row is postcontrast axial T1W images (g and h), which show no enhancement in lesions and no abnormal signal intensity can be detected in cervical cord.

## 3. Discussion

MS is usually diagnosed by demonstrating clinical and/or radiographic evidence of dissemination of disease in time and space [11, 12]. Although the diagnosis of classic multiple sclerosis generally does not require surgical intervention, some cases pose considerable diagnostic difficulty and may require brain biopsy of which large demyelinating lesions resembling brain tumors is an example. The occurrence of tumor-like demyelination is reportedly rare, being estimated at 1-2/1000 cases of multiple sclerosis [13]. It clinically presents with headache, cognitive abnormalities, mental confusion, aphasia, apraxia, motor symptoms, and/or seizure [14].

Generally, TMS is defined as a solitary intracranial lesion larger than 2.0 cm in diameter, but multiple lesions are not uncommon [14]. Although MRI has increased our ability to highlight MS lesions, it often fails to provide an unambiguous diagnosis. This is particularly true when the lesions present as large, space-occupying lesions misinterpreted as tumor, abscess, or infarct [15]. This may lead to inadvertent brain irradiation or surgery. However, some MRI features are more suggestive of TMS. These include incomplete rim enhancement, mixed T2-weithed iso-and hyper-intensity of enhanced regions, absence of a mass effect, and absence of cortical involvement [16]. Our cases presented with an acute sever neurological illness accompanied by papillitis, motor symptoms, seizure, positive OCB in CSF, and prolonged VEP and SEP. The MRI showed contradictory findings with respect to the diagnosis of TMS. On one hand, multiple large round-shaped lesions were accompanied be extensive prelesional edema, involvement of splenium of corpus callosum, and heterogenous enhancement all in contrast to more common findings in TMS. On the other hand, there was no clear cerebral mass effect though the latter also may be explained by bilaterality of lesions. Since biopsy was denied by patient and parents, an oncology consultation was made and on the basis of the presence of papillitis, positive OCB, and in spite of seizure which is not a common presentation of early MS and some atypical features of MRI we decided to proceed with a short course of steroid as trial therapy. Fortunately, fast improvement of symptoms suggested that we were dealing with a TMS, and this was confirmed by clinical and MRI followup of the patient. This scenario will suggest that when we are dealing with a patient with atypical clinical and MRI presentation highly suspicious for TMS, and there is an obstacle for brain biopsy: a short course of steroid therapy and followup including imaging may play a role in clarifying the diagnosis and leading to correct root of therapy.

## 4. Conclusion

TMS is an uncommon diagnostic challenge. In TMS cases, the correct diagnosis is very worthy for eliminating biopsy, unnecessary radiotherapy, and execution of early treatment. Although developed new and advanced methods of imaging can distinguish TMS from other differential diagnosis, we still believe that clinical findings and physician suspicion play a crucial role in diagnosis, and when there is an obstacle to brain biopsy, a trial therapy with steroid may play a promising role in this setting.
